# Draft genome sequence of *Bacillus pumilus* strain EZ-C07 isolated from digested agricultural wastes

**DOI:** 10.1186/s13104-018-3710-1

**Published:** 2018-08-22

**Authors:** Elvira E. Ziganshina, Waleed S. Mohammed, Elena I. Shagimardanova, Leyla H. Shigapova, Ayrat M. Ziganshin

**Affiliations:** 1 Department of Microbiology, Institute of Fundamental Medicine and Biology, Kazan (Volga Region) Federal University, Kremlyovskaya str. 18, Kazan, 420008 Russia; 2 Laboratory of Extreme Biology, Institute of Fundamental Medicine and Biology, Kazan (Volga Region) Federal University, Kazan, 420021 Russia

**Keywords:** Poultry manure, Bioreactor, Genome sequencing, *Firmicutes*, *Bacillus pumilus*

## Abstract

**Objectives:**

*Bacillus* species, belonging to the family *Bacillaceae*, are rod-shaped aerobic or facultative anaerobic Gram-positive bacteria that can be isolated from various environmental niches. *Bacillus pumilus* strains are resistant to unfavorable conditions such as UV, H_2_O_2_ and chemical disinfection. Furthermore, *B. pumilus* strains synthesize multifarious important enzymes and can be used in the production of some fermented foods, bioremediation of wastewater systems and biodegradation of environmental contaminants. Hence, investigation at the genomic level is required to understand their ecology, genetics and potential applications.

**Data description:**

In this research, we provide the genomic insights into one *Bacillus* species (EZ-C07) isolated from digested agricultural waste materials. The draft genome of the strain EZ-C07 consists of 3,724,869 bp with 3890 coding sequences and 41.5% G + C content. Based on 16S rRNA gene sequence analysis followed by in silico DNA–DNA hybridization studies, the strain EZ-C07 was identified as *Bacillus pumilus* belonging to the family *Bacillaceae* within the phylum *Firmicutes*. The whole genome shotgun project of *B. pumilus* strain EZ-C07 can be accessed at DDBJ/ENA/GenBank under the Accession QLVI00000000.

## Objective

*Bacillus* species, belonging to the family *Bacillaceae*, are rod-shaped aerobic or facultative anaerobic Gram-positive bacteria that can be isolated from various environments. One of the features of this genus is the ability to form endospores in response to various environmental and nutritional stresses [1, 2]. 16S rRNA gene sequence analysis showed a high level of phylogenetic heterogeneity in the genus *Bacillus*. Members of this genus can produce a wide range of useful pharmaceutical, agricultural and industrial products (such as antibiotics, enzymes, amino acids, sugars) [1–3]. Also, several *Bacillus* strains with strong proteolytic activities displayed the robust survival in the protein-fed anaerobic biogas reactors and finally improved the biogas productivity [4]. The anaerobic digestion of biomass belongs to a more suitable method to utilize various agricultural waste materials [5, 6].

From the array of various *Bacillus* species, *Bacillus pumilus* strains are resistant to unfavorable conditions, including UV, H_2_O_2_ and chemical disinfection [7, 8]. In addition, based on their abilities to synthesize multifarious enzymes and several other bioactive compounds [9, 10], *B. pumilus* strains can be used in fermented foods production [11], biofertilizers synthesis [12], bioremediation of wastewater systems [13] and biodegradation of environmental contaminants [14]. Moreover, some *B. pumilus* strains were previously isolated from a biogas reactor utilizing abattoir waste [15]. Hence, more investigations at the genomic level are required to understand ecology, genetics and potential applications of *B. pumilus* strains in different biotechnologies. The search of various useful bacterial strains is a thrust area in biotechnological research and their implications to produce various compounds.

## Data description

*Bacillus pumilus* strain EZ-C07 was isolated from the effluent of a biogas reactor fed with agricultural wastes, including chicken manure, and operated at high ammonia levels (> 4.0 NH_4_–N g L^−1^). The chicken manure was obtained from a poultry farm located in the Zelenodolsky district, the Republic of Tatarstan (Russian Federation). The bacterial strain *B. pumilus* EZ-C07 was cultured on Luria–Bertani agar at + 37 °C for 24–48 h under microaerophilic conditions. Genomic DNA extraction and preparation for further sequencing were performed as described previously [16]. Briefly, genomic DNA of the strain EZ-C07 was isolated by using a FastDNA spin kit (#116540600; MP Biomedicals, USA) and a FastPrep-24 homogenizer (#116004500; MP Biomedicals, USA). Extracted DNA quality was estimated by agarose gel electrophoresis, while concentration and purity of the received DNA were estimated with a NanoDrop 2000 spectrophotometer (#ND-2000; Thermo Fisher Scientific, USA). Extracted DNA sample of the strain was stored at − 20 °C until further processing. The identification of  the strain *B. pumilus* EZ-C07 was based on morphological characteristics and biochemical tests and then confirmed by sequencing of its 16S rRNA gene (Accession Number MH510687) on an ABI PRISM 3130xl Genetic Analyzer (#4359571; Thermo Fisher Scientific, USA) (Table 1).

**Table 1:** Overview of data files/data sets

Label	Name of data file/data set	File types (file extension)	Data repository and identifier (DOI or Accession Number)
Data file 1	Whole genome shotgun project	FASTA	DDBJ/ENA/GenBank (Accession QLVI00000000)
Data file 2	16S rRNA gene sequence	FASTA	GenBank (Accession MH510687)

To perform genome sequencing, DNA was fragmented with a Q800R2 DNA Shearing Sonicator (#Q800R2-110; Qsonica, USA), and DNA library preparation was fulfilled with a NEBNext Ultra DNA Library Prep Kit for Illumina (#E7370S; New England Biolabs Inc., USA). Effectiveness of DNA fragmentation and preparation of DNA library was analyzed with a 2100 Bioanalyzer Instrument (#G2939BA; Agilent Technologies, USA). The bacterial genome of *B. pumilus* EZ-C07 was sequenced by a HiSeq 2500 Sequencing System (SY–401–2501; Illumina, USA), HiSeq PE Rapid Cluster Kit v2 (PE-402-4002; Illumina, USA) and HiSeq Rapid SBS Kit v2 (500 cycles) (FC-402-4023; Illumina, USA). Sequence quality of the genome was analyzed with FastQC software version 0.11.7. The genome was then assembled with Velvet version 1.2.10 [17], and the received contigs were ordered with Mauve version 2.4.0 [18] with default parameters. The genome sequence of *B. pumilus* EZ-C07 was annotated with the RAST automatic annotation server [19]. The rRNA and tRNA genes were determined with the RNAmmer 1.2 server [20] and tRNA scan-SE 1.23 search server [21], accordingly. Finally, based on 16S rRNA gene sequence analysis followed by in silico DNA–DNA hybridization studies (against type strain *B. pumilus* ATCC 7061), the strain was identified as *Bacillus pumilus* belonging to family *Bacillaceae* within the phylum *Firmicutes*.

The obtained genome sequence of *B. pumilus* EZ-C07 included 31 contigs (> 500 bp in size) with a calculated size of 3,724,869 bp in length and N50 of 183,828 bp. The G + C content for the draft genome is 41.5%. A total of 3890 coding sequences (CDS) were predicted, where 1818 CDS (47%) were annotated as seed subsystem features and 2072 CDS (53%) were annotated as outside of the seed subsystem. In total, 2675 and 1215 proteins were identified as non-hypothetical and hypothetical, respectively. It was demonstrated that genome encoded at least 3 rRNAs and 50 tRNAs. The strain *B. pumilus* EZ-C07 possesses numerous genes involved in monosaccharides and proteins metabolism, fermentation, as well as biphenyl and gentisate biodegradation. Many genes responsible for the bacterial strain resistance to several antibiotics and various toxic compounds, such as arsenic, cobalt, zinc and cadmium, were also identified. This resistant bacterial strain can be used in different biotechnologies.

## Limitations

The obtained results are based on the draft genome assembly; therefore, the exact genome’s length, number of rRNAs, repetitive elements cannot be certainly reported. Also, the presence of any plasmids cannot be clearly predicted.
